# Mesenchymal Stem Cells from Adipose Tissue in Clinical Applications for Dermatological Indications and Skin Aging

**DOI:** 10.3390/ijms18010208

**Published:** 2017-01-20

**Authors:** Meenakshi Gaur, Marek Dobke, Victoria V. Lunyak

**Affiliations:** 1 Aelan Cell Technology, San Francisco, CA 94107, USA; mgaur@aelanct.com; 2 Division of Plastic Surgery, University of California, San Diego, La Jolla, CA 92121, USA; mdobke@ucsd.edu

**Keywords:** adipose-derived stem cells, skin homeostasis, dermis, epidermis, subcutaneous adipose depot, matrikines, aging, wound healing, clinical applications

## Abstract

Operating at multiple levels of control, mesenchymal stem cells from adipose tissue (ADSCs) communicate with organ systems to adjust immune response, provide signals for differentiation, migration, enzymatic reactions, and to equilibrate the regenerative demands of balanced tissue homeostasis. The identification of the mechanisms by which ADSCs accomplish these functions for dermatological rejuvenation and wound healing has great potential to identify novel targets for the treatment of disorders and combat aging. Herein, we review new insights into the role of adipose-derived stem cells in the maintenance of dermal and epidermal homeostasis, and recent advances in clinical applications of ADSCs related to dermatology.

## 1. Introduction

The skin is a vital organ that functions not only as a protective barrier against environmental factors, but also in the synthesis, processing and metabolism of structural biomolecules such as lipids, proteins and glycans, and in the production and secretion of hormones. Skin aging is a complex biological process that becomes evident through the individual’s appearance and is caused by intrinsic factors such as structural and hormonal, and by environmental/extrinsic factors such as UV (longwavelength ultraviolet radiations (UVA) and medium wavelength ultraviolet radiations (UVB) exposure) radiations and chemicals [1,2]. Dermatological aging is driven by deficiencies in (a) important intrinsic cellular processes such as DNA repair and stability; mitochondrial function; cellular control over cell cycle and apoptosis; cellular metabolism; as well as (b) progressive loss of epidermal homeostasis such as the integrity of the extracellular matrix; changes in hormonal levels and the ability of complex cellular systems to provide communication between all of the counterparts. The physiological changes associated with aging of the skin are manifested in xerosis, dramatic loss of skin elasticity due to damage to collagen and elastin fibers; as well as barrier function, striae, modification of rhytides and deficiencies in the regenerative property of the skin (efficient turnover of epidermal cells) all of which ultimately result in thinning of the skin, malar fat atrophy and pigmentary changes [2,3].

The regenerative property of skin is essential for maintenance of the skin barrier during normal developmental process and for wound healing. Impaired skin regeneration results in debilitating effects on the organism in general and is a leading cause of a number of pathological conditions. The regenerative ability of skin is maintained by the stem cells (SCs) that are present in the skin to provide for cellular turn over during skin homeostasis and repair upon injury [4,5]. Whereas the morphogenetic changes that are associated with SC-driven regeneration and orchestrated cell stratification are intensively investigated, the underlying molecular mechanisms which execute such complex decision-making processes such as cell proliferation, differentiation and migration within the epidermis remain poorly understood.

Interplay between multiple signaling pathways on genetic and epigenetic levels seems to be important for regenerative properties of the skin SCs [6,7,8,9,10]. During adult skin homeostasis, the resident SCs from the different stem cell niches, including the hair follicle (HF), interfollicular epidermis [11] and sebaceous glands (SGs) have been proven to be important in the maintenance of healthy dermis and epidermis. In addition to SCs, other types of epidermal progenitors have come to the light in recent investigations, suggesting no less complex mesenchymal-epithelial crosstalk that governs skin regeneration through the secreted stimulatory factors: cytokines, chemotactic factors extracellular matrix (ECM) remodelers, growth factors and their inhibitors [12,13].

In parallel, recent clinical research points to the fact that adult stem cells of mesenchymal origin from the perivascular adipose depots can be successfully deployed for the treatment of several skin conditions. They can boost skin regeneration after injuries and those due to natural aging-related decline [14]. In this review, we will discuss the applications of adipose–derived stem cells ADSCs aimed for more efficient re-epithelialization and highlight the current view on the cascade of the factors essential for skin regeneration, many of which are secreted by these cells. We will touch upon the complex and the hierarchical relations between ADSCs and numerous reservoirs of long-term SCs and progenitors found to reside in the skin epithelium, as well as review the recent conceptual paradigms aimed to explain how these interactions modulate SC niches in a remarkably orchestrated fashion to allow different resident niches to be appropriately fueled during skin homeostasis and how interruptions in the spatial and temporal order of these events result in pathological conditions. Current research has raised many new fascinating questions and has unraveled new and exciting paradigms through which one can seek an understanding of how aging influences a number of the functional relationships important for skin regeneration and repair. We will attempt to render these new ideas in this manuscript.

## 2. Skin Structure and Cellular Composition as It Relates to Regenerative Medicine

**Figure 1:**
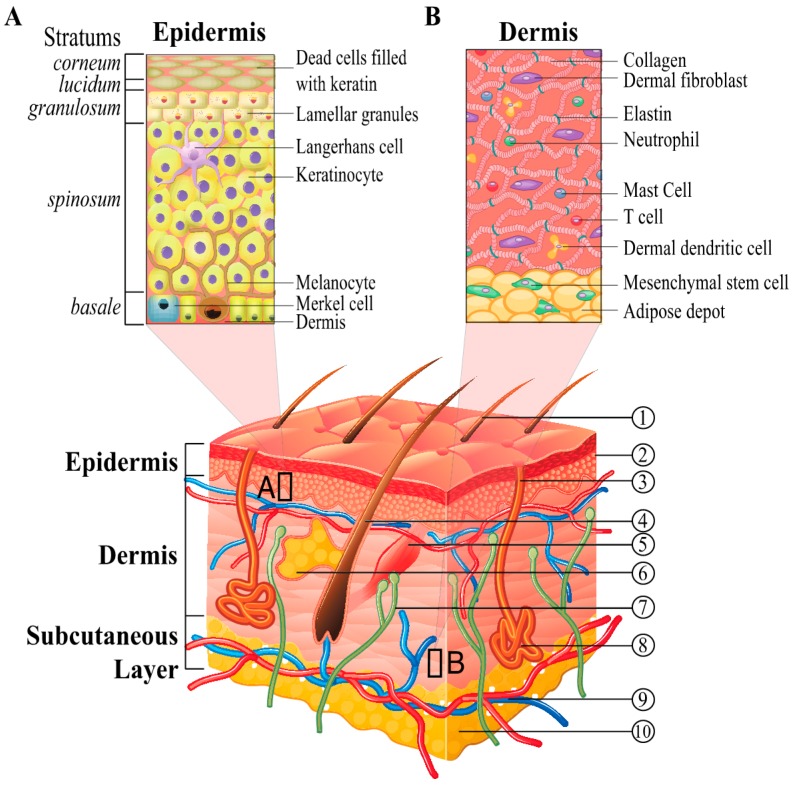
Skin Structure. The skin is composed of 3 main layers-Epidermis, Dermis and Subcutaneous layers containing (1) hair shaft; (2) stratum corneum; (3) sweat-pore; (4) hair follicle; (5) arrector pili muscle; (6) sebaceous gland; (7) nerve; (8) eccrine sweat gland; (9) cutaneous vascular plexes; (10) adipose depot. Detailed structure of (**A**) epidermis is shown with the stratum layers and (**B**) cellular composition and dermis is shown.

Before we dive into the complexity of biological pathways governing skin regeneration and repair, let us take a look into the structure and composition of the skin. The human skin is the largest organ in the body that performs numerous indispensible physiological functions, such as: protection from exogenous chemical, physical, immune and pathogen insults; defense against free radical and UV radiation; major participation in thermoregulation and thermoisolation; and, importantly, is the organ entrusted with significant endocrine function burden (vitamin D synthesis, peripheral conversion of pro-hormones) [15]. Its thickness varies from 0.05 to 2 mm and is composed of four main layers shown in Figure 1.

The external, highly organized structure, *stratum corneum*, 10–20 μm thick, is highly hydrophobic and contains 10–15 layers of interdigitated dead cells called corneocytes. It is a major barrier to the penetration of environmental factors, as well as “active ingredients” included in cosmetic and dermatological products. Often defined as a “brick and mortar” structure, the corneocytes of hydrated keratin comprise the “bricks”; and multiple lipid bilayers semicrystalline composed of fatty acids, ceramides, cholesterol and cholesterol esters are viewed as a “mortar” holding the structure [16,17]. Skin penetration by topical agents occurs via this intercellular microroute and numerous techniques exist to provide for temporary disruption of this highly organized structure [18] shown in Figure 1A.

**Figure 2:**
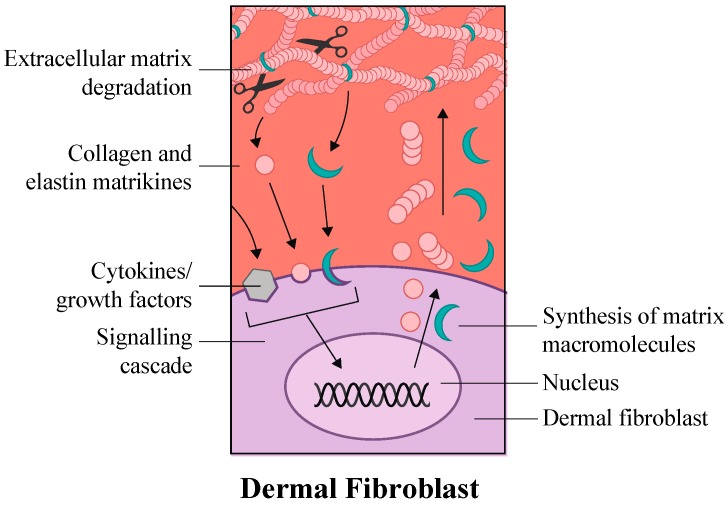
A Schematic representation of dermal fibroblast in regulation of skin homeostasis. Fibroblasts have been shown to influence the signaling pathways for the synthesis of extracellular matrix (ECM), collagen and growth factors.

The second outermost layer is viable *epidermis*, which originates from the ectoderm during embryonic development (Figure 1) [19,20]. It is (~100–150 μm thick) and is often divided into *stratum lucidium*, *stratum granulosm*, *stratum spinosum*, and *stratum germinatevum* (*basale*). All these sub-layers are composed of keratinocytes at various stages of differentiation. Keratinocyte is the predominant cell type in the epidermis, constituting 95% of the cells. The keratinocytes found in the basal layer (*stratum germinatevum*) of the epidermis are sometimes referred to as “basal cells” or “basal keratinocytes”. In addition to the keratinocytes, numerous other cell types can be found in the epidermis (dendritic T cells, adipose cells, melanocytes, Merkel cells, Langerhans cells, and epidermotropic lymphocytes). Numerous active catabolic enzymes including, but not limited to, lipases, phosphatases, esterases, nucleotidases and proteases are present in the intercellular space as shown in Figure 2 and reviewed in [19]. As mesenchymal cells populate the skin, they transmit signals that instruct the stratification of the epidermis during development and dictate the positioning of downgrowths that mark the initiation of hair follicle (HF) morphogenesis [21]. The epidermis is subjected to homeostatic regulation during maturation as the *stratum basale* cells undergo mitotic proliferation during which the daughter cells move outwards up from basal to suprabasal to *spinosum*. The migrating cells change their morphology as they undergo multiple stages of cell differentiation due to deployment of dedicated transcriptional programs. Upon migration and entry of the spinous layer, the cellular genome undergoes changes in its transcriptional programs. For instance, the expression of genes encoding keratin 5 and 14 (*KRT5*/*K5* and *KRT14*) is turned off, and the transcriptional upregulation of genes encoding *KRT1* and *KRT10* is turned on. This switch in the keratin production is viewed to be instrumental to forging a robust, interlinked with desmosomes an intermediate filament (IF) proteins network [22]. This IF network is viewed as a resilient structural scaffold aimed to reinforce cell–cell junctions and provide a resistance against mechanical stresses. At the late stage of terminal differentiation, granular cells express structural proteins that, when cross-linked, form structural 3-D support for lipid bilayers that are extruded from intracellular lamellar granules into the extra-cellular space between squamous (dead *stratum corneum* cells). This process may be viewed as “waterproofing” or sealing the skin surface. Eventually terminally differentiated cells shed from the *stratum corneum* surface (desquamation).

The innermost basement membrane layer is rich in extracellular matrix (ECM) proteins and growth factors. The formation of this layer takes its humble beginnings as a collaboration between the mesenchyme and stratifying epidermis, both of which participate in the production and organization of the basement membrane. The basement membrane that is attached to the epidermis provides a physical boundary between the epithelium and the third layer of the skin, *dermis*. It also serves as “a provision support center” providing growth-promoting factors, enzymes and structural components. The structural composition of the dermis is saturated with nerves, blood vessels, hair follicles, and sebaceous and sweat glands (shown in Figure 1). The presence of various lineages of dermal fibroblasts is important for manufacturing and supporting the polymerization of collagen and elastin fibers comprising ECM, a structure that provides for the elasticity of the dermis (Figure 2). Dermal adipose cells, together with mast cells and infiltrating leukocytes, are also present in this skin layer and shown in Figure 1B. Skin homeostasis and remarkable resilience of the skin is provided by SCs, which reside in different niches of the skin. Recent studies have begun to explain the mysteries behind these special “fountains of youth” (for extensive review [4]).

The last layer, the hypodermis or subcutaneous layer, is encompassed by adipose cells, mesenchymal stem (stromal) cells (MSCs), blood and lymph vessels (Figure 1). Numerous studies demonstrate that adipose tissue provides a remarkable influence on the microenvironment by the secretion of a wide-range of bioactive factors with a diverse set of functions. This includes lipid metabolism, energy balance and insulin sensitivity, which also have proven to be important in the regulation of angiogenesis, immunomodulation and inflammatory response. The secretory repertoire of adipose tissue can influence tissue and organ homeostasis at the autocrine, paracrine and/or endocrine, and gene expression level [23]. In addition, it has been reported that mesenchymal stem cells contain 500 times higher per gram of subcutaneous adipose tissue than bone marrow [24,25,26,27]. A complex and interconnected relationship has been shown between hematopoietic cells, fibroblasts and keratinocytes during skin homeostasis; and yet the contribution of the SC from subcutaneous and intradermal adipose tissue depots and intradermal adipocytes themselves have just recently been investigated despite the intensive use of these cells in plastic and reconstructive surgery [28,29,30,31]. Functional analysis of rodent models demonstrate that adipose depots are involved in mediation of the communication among multiple cell types, as well as in coordination and management of keratinocyte and fibroblast proliferation and migration in order to ensure epidermal and dermal repair [12,32,33]. Consequently, the beneficial therapeutic effects in the intradermal and subcutaneous adipose compartments could be attributed to the capacity of resident mesenchymal SCs, not only to heal sites of inflammation and injury, but also for their ability to secrete numerous pro-regenerative, anti-fibrotic and anti-apoptotic signaling molecular and growth factors integral to endogenous repair processes [34,35]. In other words, adipose tissue-resident mesenchymal stem cells could well be viewed as “endogenous factories” producing trophic mediators able to support all of the functional skin layers to ensure skin homeostasis, regeneration and repair.

## 3. Dermatological Aging and Practical Use and Significance of Non-Invasive Methods for Its Assessment

Although dermatological aging is a natural process that is caused and modified by intrinsic and external factors, it inevitably leads to changes that are physical (appearance), structural, and physiological, affecting barrier function and neurosensory perception. The key structural changes are caused by proteins, glycosaminoglycan, water, and lipids; and the structural modifications in these biological molecules have been reviewed previously [36,37,38]. Structurally, aging skin undergoes thinning of the epidermis that is caused by the reduction of vascularity and hydration without change in the number of epidermal layers [39,40]. On average, the thickness of the epidermis is reduced by about 6.4% during each decade of aging and there is a decreased number of mast cells and fibroblasts [40,41,42]. The thinning may also be caused by the decrease in the keratinocyte proliferation rate and by the reduction of the glycoprotein levels; for example, epidermal transmembrane keratinocyte glycoprotein. CD44 positive cells have been reported to have a regulatory role in proliferation of keratinocytes and in the maintenance of hyaluronic acid homeostasis decreases with aging [43,44]. Decreased production of collagen, elastin, glycosaminoglycans and hyaluronic acid due to the reduction in total fibroblasts leads to the thinning of dermis [1]. The extracellular matrix (ECM) changes associated with aging have also been attributed to circulating sugars and glycation end-products (AGEs). AGEs are generated as a result of non-enzymatic glycation of amino groups on proteins by reducing sugars, following the Maillard reaction, impacting on the changes in ECM structures [45,46]. AGEs are believed to activate the downstream signaling pathways responsible for increased oxidative stress and inflammation ultimately leading to tissue damage and a number of the diseases associated with aging that have been reviewed in detail [47]. Structural changes in the dermis occur because of a higher degree of calcification in the aged skin, degradation of elastin fibers, and changes in the organization of collagen bundles as they become disorganized with aging [48]. These ECM molecules are critical for providing structural support and in the regulation of signaling pathways for normal growth and development of skin cells, as well as during wound healing.

Skin aging effects are external as well as intrinsic, but they can be monitored/measured based on the skin appearance: texture and roughness, fine lines and wrinkles, structure, elasticity, hydration and barrier function. Many new non-invasive or minimally invasive bioengineering advances in recent years have enabled the quantitative analysis of skin properties during the aging process [49,50,51]. Trans-epidermal water loss (TEWL) is widely used for measuring the changes in the skin barrier integrity [51,52]. TEWL is defined as the measurement of the water that passes through the epidermis to the surrounding area by diffusion and evaporation. Skin corneometer measures electrical capacitance of skin, thereby measuring the extent of stratum corneum hydration [52]. Confocal Raman spectroscopy has been used extensively to measure the in vivo skin penetration of biological molecules, such as retinol, as it allows for the quantitation of water and other molecules in the skin [53,54]. This sophisticated technique relies on the Raman (inelastic) of monochromatic light and presents a highly detailed spatial resolution. Videomicroscopy and Fringe Projection are non-contact quantitative techniques for skin surface changes, while the Dermal Torque meter, Ballistometer and Cutometer are used for measuring skin firmness and elasticity [52,55]. Recently, Hahn et al. 2016, measured skin wrinkles by PRIMOS Lite 3D Face and Skin Scanner Analyzing System and used the DermaLab USB elasticity probe (Cortex Technology ApS, Hadsund, Denmark) for skin elasticity measurement [50].

The latest non-invasive non-contact techniques such as phase-shift rapid in vivo measurement of skin (PRIMOS) and surface evaluation of living skin (SELS) using Visioscan VC 98 rely on the use of optical systems for directly measuring skin roughness [56,57]. These systems are easy and time efficient as compared to the techniques that needed skin replica to be generated. Recent studies compared the data obtained using PRIMOS and Visioscan VC98 and concluded that Visioscan VC98 measurements generated highly reliable and consistent results for the parameters measured [56,57].

In addition, developments of novel devices made it possible to evaluate not only skin morphology but also molecular processes within the skin by noninvasive instrumental measuring techniques [26,40,51,52,55,58,59]. Raman spectroscopy is a non-destructive analytical method for analysis of molecular compounds [60]. Using this technique, Gniadecka, et al. [60] compared changes in the collagen properties such as fragmentation, thickening, solubility and arrangement of collagen fibers in photoaged vs. chronologically aged skin. These techniques can be successfully applied to quantify and objectively assess the efficacy of the products or procedures, as well as to decipher important signaling pathways important for skin homeostasis.

Next, we will discuss the signaling pathways that outline the complexity of the balanced interactions within skin infrastructure and its impact on derma aging.

## 4. Signaling Pathways of Cellular Interactions within Skin

All major cells such as keratinocytes, fibroblasts, macrophages, adipocytes and SCs within the skin layers have a capacity to communicate between themselves and interact with their environment. The internal and external stimuli can trigger the complex changes in these cell-to-cell communication pathways to orchestrate the appropriate responses [61]. The skin cell systems use several clearly defined signaling pathways that will transfer information from the cell surface to the genome to facilitate gene activities.

Mouse genetics studies have uncovered multiple signaling pathways of great importance for different events related to skin homeostasis. The complexity of skin differentiation and epidermal stratification, wound healing and regeneration in development and disease settings, is orchestrated by multiple spatially and temporarily coordinated pathways including Wnt, Notch, mitogen-activated protein kinase (MAPK), nuclear factor-κB (NF-κB) and integrin-linked kinases. Numerous transcriptional factors that drive developmental cascades and cellular viability transcriptional programs seem to be enlisted to cross-communicate the instructions of the signaling pathways to the gene targets, such as transcriptional regulators p63 (which is related to p53), the AP2 family, the CCAAT/enhancer-binding protein (C/EBP), Kruppel-like factor 4 (KLF4), transforming growth factor (TGF-β) activation and numerous others [10,21,62,63,64,65,66].

The interplay between critical signaling pathways, gene transcription and the production of growth factors imposes very specific influences (instructions) on heterogeneous cell compositions in normal and pathological settings. The full complexity of these elaborate cascades is just beginning to emerge. In particular, increased understanding of the role of growth factors, cytokines and matrikines in skin rejuvenation and regeneration, wound repair, and reversal of photo-aging has provoked a great deal of interest, resulting in numerous studies aimed to evaluate their role in repair, regeneration and remodeling of dermal infrastructure. A large number of cosmeceutical products include in their composition either a single or combination of multiple human growth factors. Cytokines and matrikines are currently marketed for skin repair and rejuvenation. The results of the clinical assessment of some of these topical products show beneficial effects in reducing the signs of facial skin aging thus supporting skin homeostasis [67,68,69,70,71,72,73,74].

We have summarized some, but not all, of the most interesting factors in Table 1. Our hope is to give the reader a high-level view of an ever-expanding realm of the inter-connective pathways and their immediate read-outs by specific and/or cohort of the targeted cells leading to important functional outcomes for skin homeostasis, regeneration and repair functions. This is essential for the critical evaluation of a plethora of emerging claims regarding regenerative dermatological therapies.

Several studies emphasized the role of interleukins and interleukin receptors in the skin. These factors and their receptors have an essential role in regulation of the inflammatory stage of wound healing and regeneration [116,117,118]. In addition, low doses of interleukins are found to have an anti-aging effect (see Table 1) and [119,120].

An intriguing example worthy of discussion in the context of this review is insulin growth factor (IGF-1) and its binding proteins (IGFBP1, IGFBP2, IGFBP3). The ratio between IGF-1 and IGFBP3, have been associated with facial aging and skin wrinkling [103]. IGF binding protein 1 (IGFBP1) also regulates the signals generated by adhesion receptors and growth receptors, thereby regulating the cellular processes. In addition, the cross talk between insulin-like growth receptor and β1-integrin receptor has been reported to play an important role in the prolidase activity and collagen biosynthesis [102], which together with IGFBP1 and 2 and their proteolytic fragments, have been shown to improve tissue repair process under inflammatory conditions, by acting upon the pathways that control proliferation and migration of HDFs [121].

Human dermal growth factors and cytokines are important for collagen, elastin and hyaluronan production [122]. Hyaluronan (HA) synthesis and regulation of its degradation is essential for maintenance of extracellular matrix homeostasis. Alteration in the expression of hyaluronic acid (HA) and its metabolizing enzymes is associated with dermatological aging of the human skin [123]. Notably, TGF-β1, bFGF, EGF, and PDGF-BB are commonly linked to enhancement of the total amount of HA production in skin fibroblasts through the targeted up-regulation of HA synthase expression [124]. It has been reported that fibroblasts treated with growth factors are capable of triggering healing and regenerative processes such as proliferation, angiogenesis and extracellular matrix component (ECM) synthesis. This results in the release of VEGF and HGF by fibroblasts and it has been reported that enhanced synthesis of type I collagen and hyaluronic acid (HA) can be accomplished in these experimental conditions [125]. In addition, recently it has been shown that VEGFR are involved in cell migration [101,126]. PDGF signaling and its receptor is involved in hair follicle morphogenesis [127].

Intriguingly, recently it has been postulated that dermal adipocytes secrete adipocytokines, such as adiponectin and leptin. Sensing these adipokines by human dermal fibroblasts expressing receptors for adiponectin and leptin results in the significant increased production of hyaluronic acid (HA) and collagen, a major extracellular matrix component (ECM) of dermis [128]. In accordance with these findings, it has been shown that leptin promotes wound healing in the skin [129]. Among these findings then another member of the TGF-β superfamily, Activin A, have shown to be involved in physiological and pathological processes associated with tissue homeostasis, cell differentiation and, ultimately, with wound healing [130].

**Figure 3:**
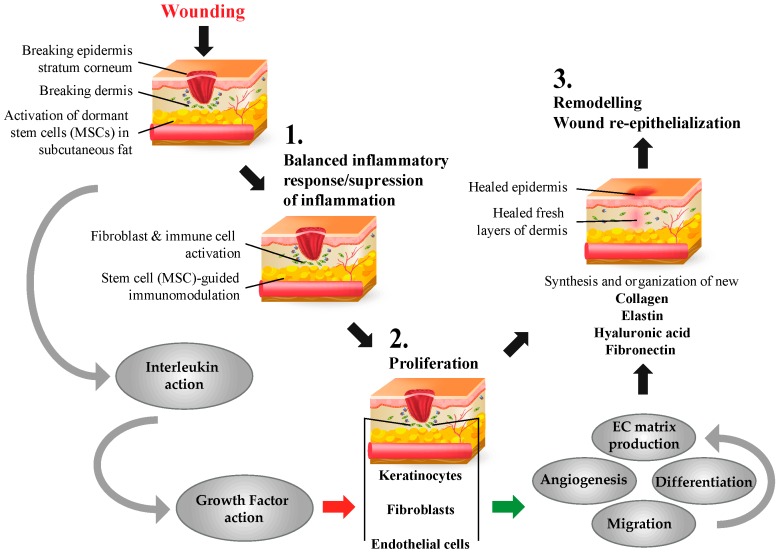
Stages of Wound Healing: Wound healing is a 3-step process. (1) Inflammation stage initiated by migration of inflammatory and stem cells to the wound-site; (2) Proliferation and Granularization stage involving secretion of growth factors and migration of fibroblasts, keratinocytes and endothelial cells to initiate ECM production and repair and; (3) Re-epithelialization stage is the final stage of wound healing process marked by the synthesis and organization of new collagen and elastin fibers resulting in healed epidermis.

Some of the listed protein factors, especially the transforming growth factor-β (TGF-β), α-granule-derived factors such as platelet-derived growth factor (PDGF), fibroblast growth factor (FGF-2/bFGF), epidermal and hepatocyte growth factors EGF and HGF, insulin-like growth factor (IGF), and angiogenic vascular endothelial growth factor (VEGF) have been commonly called skin matrikines and assigned a functional significance in wound-healing and/or in skin remodeling and regeneration [131,132]. Matrikines themselves or their hydrolytic peptides can link a matching cell-surface receptor to a transducer in order to relay information into the cell(s) using various signaling pathways. For example, TGF-β-PI3K axis that governs collagen-1 production (shown in Figure 3)—to be discussed below. In simplistic terms, each matrikine works like a key to a keyhole. Different matrikines can “unlock different doors”, relay a different messages to cell systems and trigger different functional outcomes.

Next we will tease apart some mechanistic aspects of these complex cascades by taking a closer look into skin wound healing process.

## 5. Wound Healing Paradigms

Skin is a self-repairing organ and maintains homeostasis through very organized yet complex mechanisms. Any injury or damage to skin results in cutaneous wounds that either heal itself or require an intervention based on the etiology. Understanding of this process of wound healing is important to tease apart critical signaling circuitry in communication of multiple cell types that coordinates proliferation, migration and factors secretion during epidermal and dermal homeostasis. While it is important to acknowledge that wound healing process is different from skin regeneration in its core, we believe that knowledge gained from its building blocks will shed light into the regenerative process itself and will empower many therapeutic approaches in medical practice for treating pathological conditions and aging.

Effective wound healing leads to the restoration of skin tissue integrity and occurs through a highly organized multistage and truly dynamic process that involves distinct stages such as (1) *inflammation*; (2) *proliferation and granularization* and; finally, (3) *re-epithelialization* (shown in Figure 3).

Wound healing process is initiated following changes in fibrin assembly at the wound site and recruitment of SCs in the skin, where functional activities will proceed through all of three distinct stages for successful wound healing [109,133,134].

The first inflammatory stage is triggered by fibrin changes and is immediately followed by hemostasis and migration of inflammatory cells and mediators of inflammatory response to the wound. This includes monocytes, neutrophils, macrophages as well as SCs from different niches, including cells from dermal and subcutaneous adipose depots. All these cells secrete growth factors and cytokines that regulate the signaling cascade and immunological pathways for wound healing [33,135,136,137]. Several studies show the role of interleukin receptors in the skin. These receptors have an essential role in the regulation of the inflammatory stage of wound healing and regeneration (see Table 1 and [116]). The natural course of wound healing at this stage involves remodeling of dermal collagen and other matrix molecules at the initial inflammatory phase, which has been reported to manifest massively high levels of matrix metalloproteinases (MMPs) that degrade the fragmented collagenous matrix [138,139,140]. Growth factors and cytokines such as TGF-β, TNF-α, PDGF, IL-1, IL-6, and IL-10 participate in the step-wise balancing act between development of inflammation and its rapid resolution [141].

Similar to above, the spatial-temporal process involving numerous growth factors and cytokines marks the transition from inflammatory phase of wound healing to the second (proliferative) granulation phase. This process engages FGFs, TGF-α, TGF-β, PDGF, HGF, EGF, IGF-1, as well as CSF, IL2, IL6, IL8, and TNF-α [29,33,79,95,106,109,110,111,142,143,144,145,146,147]. Several transmembrane adhesion proteins have been shown to be expressed at this stage. For example, CXCL16 is a transmembrane adhesion protein and is expressed by epidermal keratinocytes of normal skin upon wound injury or photodamage [148] and CXCL10 was demonstrated to have a role in the production of type I collagen, elastin and hyaluronan in dermal fibroblasts [122]. Granularization stage/proliferative stage is initiated by the proliferation, accumulation and migration of fibroblasts and epidermal keratinocytes, which are guided by the presence of environment growth factors and cytokines. The fibroblasts and keratinocytes themselves also secrete intricate cascades of matrikines. Multiple metabolic pathways lead to the formation of new collagen and to the repair of extracellular matrix during the granulation phase. Many cell adhesion molecules have recently been proven to be important modulators of fibroblast, keratinocytes and immune cell migration and coordination of their function by providing the EMC communication network in skin at this stage. For instance, the immunoglobulin superfamily glycoprotein, activated leukocyte cell adhesion molecule (ALCAM), has been implicated in the processes of cell adhesion and migration, and higher levels of ALCAM promote wound healing [149]. VE-Cad, an adhesion molecule of intracellular junction, is required for angiogenesis and has been shown to be associated with VEGF binding pathways [150,151]. This stage is marked not only by substantial and extended production of new undamaged collagen, but also by initiation of angiogenesis at the wound site resulting in new blood vessel formation. Studies in rodents and other models have reported that expression of the related tyrosine kinase with immunoglobulin and epidermal growth factor homology (Tie)-1 and -2 receptors, Ang-1 and -2, as well as FGFs, VEGFs, are important regulators of the angiogenesis [152,153,154,155]. That stage is followed by the epithelialization process by keratinocytes [156].

The final third stage of wound healing is the re-modeling phase. It is a prolonged phase that eventually results in gaining the skin’s integrity and properties. The transition from granulation to wound re-epithelialization marks the beginning of dermal tissue remodeling. It is associated with skin peeling. During this stage, the intermediate ECM structure composed of low strength, unorganized type III collagen and elastin become replaced by the ECM comprising of stronger type III collagen and structured elastin fibers. This event reconstitutes strength and resiliency of the dermis and provides for its elasticity. This phase of wound healing relies mainly on keratinocytes and how well they execute their functional potential. Surface receptors for many growth factors and cytokines expressed by keratinocytes make them capable of receiving signaling messages from multiple mediators such as KGF (FGF7), TGF-β, TNF-α, EGF, IFN-γ, as well as GM-CSF and IL-1 [156,157,158,159,160,161]. Scientific explorations of this stage of wound-healing strongly suggest the presence of a double paracrine loop where keratinocytes initially stimulate fibroblasts to synthesize growth factors and then this production of the growth factors burst triggers robust keratinocyte proliferation. Proliferation of keratinocytes further amplifies initial growth factors synthesis in fibroblasts. [162,163]. This stage of wound healing could be macroscopically (wound closure) and microscopically assessed by monitoring re-epithelialization, dermal-epidermal junction, skin appendage regeneration, granulation tissue, leukocyte infiltration, and density of dermal collagen fibers.

This last remodeling phase can last for several months and, in adults, usually leads to developing a non-functioning mass of fibrotic tissue known as a scar. This contrasts the early gestation in organism development, where the injured fetal tissues can be completely recreated without fibrosis due to skin regeneration. Deciphering differences in biological pathways guarding wound healing and regeneration has tremendous potential to improve therapeutic stem cells regenerative approaches to dermatological pathologies and boost aesthetic methods aimed to reverse the visible effects of skin aging [67].

Failure of one or several stages in the wound healing processes is generally linked to vascular disease, diabetes and many other clinical conditions that are all frequently associated with healing pathologies and ultimately aging.

Next, we will discuss one of the most promising and undoubtedly popular modern day sources used in clinical dermatology-adipose-derived stem cells to counteract this and several other pathologies.

## 6. From Lipoaspirate to Adipose-Derived Stem Cells (ADSCs)

Following the discovery that adipose tissue is a rich source of adult mesenchymal stem cells that exhibit regenerative potential [164] and wound healing properties [133,165,166], the lipoaspirated tissue began to be widely used in clinical applications in autologous or allogeneic fashion. Lipoaspirate is harvested by using tumescent abdominal liposuction techniques such as power assisted liposuction, surgical resection and laser-assisted liposuction [167]. In addition, the lipoaspirate harvested after surgery undergoes further processing using enzymatic [168,169] or mechanical cell separation [170,171,172]. Most SVF isolation protocols contain the following steps: washing the lipoaspirate, collagenase enzyme treatment, centrifugation, and removal of red blood cells [171]. After decantation or centrifugation, the processed lipoaspirate composed of the structural and stromal vascular cells, called stromal vascular fraction (SVF), can also be used for grafting. SVF contains a mixed composition of cells including pre-adipocytes, adipocytes, macrophages, endothelial progenitor cells (EPCs) and adipose-derived stem cells (ADSCs) [168,173,174] and has been shown to be a rich source of growth factors such as bFGF, IGF-1, VEGF, and PDGF-BB [175]. Despite the extensive and widespread use of adipose tissue in clinical applications, there is no single standard protocol available for isolation and preparation of adipose tissue. Recent reports have highlighted the differences in the protocols for harvesting, processing, and techniques for injections among clinicians and the challenges it introduces during comparison of the outcomes of clinical therapies [176]. A recent review article comparing some of the variables in isolation techniques reported no significant differences in delivery of the therapeutic benefits that depend on the donor fat site collection choice or pre-operational site preparation, fat harvesting methods, centrifugation speed or cannula size following the use of tumescent solution. However, a better comparison strategy should be deployed to substantiate such conclusions. For instance, it was demonstrated that better retention of the graft was reported in clinical studies that used centrifugation rather than sedimentation [177]. However, use of collagenase during SVF isolation steps introduces an additional regulatory burden. SVF and subsequent isolation of ADSCs from SVF is considered “more than minimal manipulation” according to U.S. Food and Drug Administration guidance documents and requires pre-market approvals of therapeutic applications of these biologic materials even for autologous cell therapies. These strict regulations are implemented due to observed variations in the composition of SVF isolated by use of different isolation protocol or devices [178,179,180]. Currently, significant effort is applied by researchers and clinicians to develop standardized methods for SVF isolation to implement quality control systems (QC) for SVF production in order to lower the regulatory barriers [168].

One of the methods to implement more stringent QC of clinical-grade material is an isolation of homogeneous population of the cells from SVF. Numerous new studies report that subsets of the cells isolated from the adipose niche (adipose-derived stem cells ADSCs) have a potent immunomodulatory activity and can robustly induce angiogenesis. It has been shown that developmental origin of ADSCs can be traced to mural cells (or pericytes) residing in the perivascular niche [181,182,183], thus explaining the remarkable capacity of ADSCs to communicate with a variety of the cell types and instruct the immune system through the paracrine signaling. It has been noted that the location of subcutaneous white adipose tissue deposits might influence stem cell recovery. For instance, in humans a much greater number of ADSCs can be recovered from the lipoaspirates of arm adipose tissue as compared with the tissue obtained from the thigh, breast, and the abdomen (reviewed in detail [184]).

The ADSCs from SVF can be expanded and passaged in culture, generating a homogeneous ADSCs population expressing the stromal surface markers similar to bone-marrow mesenchymal stem cells (BM-MSCs): CD29, CD44, CD73, CD90, and CD105 while staying negative for hematopoietic lineage markers CD31, CD34 and CD45 [185]. The secretory properties, cell surface expression markers, multipotent potential, and immunomodulatory properties of isolated ADSC and BM-MSCs have been rigorously compared and reported previously [11,23,186,187,188] concluding that the ADSCs, by and large, display secretory properties similar to those reported for BM-MSCs. However, it is important to note that prolonged ex vivo culturing of ADSCs (often required to create enough material for clinical application) results in significant and measurable changes in ADSCs due to the process of replicative senescence [23,189,190]. Senescence involves a coordination of diverse cellular processes ranging from inflammatory signaling, metabolic and cytoskeletal changes, ability of cells to cope with DNA damage, and dramatic changes in the regulatory properties of chromatin [23,189]. We and others have previously reported that isolated and ex vivo cultured hADSCs exhibit consistent self-renewing and, upon approaching replicative senescence, cultures accumulate large non-dividing cells expressing the lysozymal enzyme, senescence-associated β-galactosidase (SA-β-Gal), manifest a loss of control for chromatin organization, activate a persistent DNA damage response (DDR) and cause robust changes in transcriptional activity [189,191,192], while preserving ADSCs phenotypical stability. These changes not only result in the reduction of differentiation potential of ADSCs, but are also associated with severe changes in ADSCs secretory repertoire impacting ADSCs paracrine function and immunomodulation. Due to the profound secretory phenotype of senescent cells, their impact on organs and tissues after transplantation is far from neutral. The role of senescence in the aging of adult stem cells is tightly linked to tissue maintenance and homeostasis and often viewed as an irreversible barrier to immortalization and tumorigenesis under the assumption that cellular senescence evolved to suppress tumorigenesis [193,194]. This view has been intensely debated in recent years [195,196]. Contrary to the assumption that senescence and tumorigenicity are always permanently connected and mutually exclusive, recent data monitoring one of the well-established markers of senescence, *p16INK4a*, in mice indicates that the activation of this hallmark of cellular senescence is, in fact, a characteristic of all emerging cancers [197], thus suggesting that cellular senescence is a quasi-stable and/or plastic cellular state prone to oncogenesis rather than a cancer preventive mechanism.

Concurrently, there are considerable ongoing clinical activities in the development of ADSCs therapeutic applications. It is important to identify not only reliable markers of senescence that will translate into human settings, but also to better understand the intimate connections between oncogenic events and senescence in order to define a set of genetic targets that are able to elicit, modulate and, most importantly, balance both of these responses. These markers would allow one to implement an additional measurable QC system to the clinical applications of ADSCs, SVF and fat grafts in general, in order to increase efficacy and safety of the clinical applications.

Given the ease in isolation in large amounts for autologous fat, SVF, or ADSCs, these materials have been widely used for a broad spectrum of clinical applications. In this review, we will limit our discussion to the applications currently in trials for indications of clinical dermatology. We will discuss some of these applications in the next section.

## 7. Adipose-Derived Stem Cells and Their Applications in Clinical Dermatology

Autologous fat graft procedures, alternatively termed as autologous fat transfer or lipo injections, deliver processed lipoaspirate containing the ECM and SVF fractions as described in the previous section. Autologous fat performs like ideal soft tissue filler and has been applied in clinical applications ranging from structural [198], facial filling and rejuvenation [199,200,201], treatment of scars [202], aging-related dermatological indications [203,204], and wound healing [165,205]. The mechanistic explanations of the significant improvements achieved in these studies are deeply imbedded in the biology of the spatial and temporal relationship between adipose tissue and different stages of wound healing. Emerging data highlight the role of cells from the adipose deposit in skin homeostasis process. It was elegantly demonstrated that adipocyte precursor cells (also called *pre*adipocytes, adipose-derived stem cells, adipose-derived stromal cells, see for details [184]) proliferate and differentiate, and mature adipocytes repopulate skin wounds following inflammatory stage of wound healing. This has been shown to occur in parallel with fibroblast migration [32]. Genetic and pharmacological ablation of adipose depots results in diminished fibroblast presence and ECM protein deposition in the regenerating dermis. All of the aforementioned defects result in long-term loss of skin homeostasis, compromising skin integrity and wound recurrence. In addition, further support was gained in studies demonstrating that stem-like cells from intradermal adipose depots, in parallel with their renewal, are necessary and sufficient to drive follicular stem cell activation [137]. Adipokines produced by adipose tissue such as adiponectin, visceral adipose tissue-derived serine protease inhibitor (vaspin) and leptin, exert hormone-like activities and can act on a systemic level [206]. These data highlight the importance of the intradermal adipogenic cells and their precursors as skin niche cells (shown in Figure 1). It also implies that adipogenic niche may alter epithelial stem cell function and have a clinical impact as a positive regulator of skin stem cell activity. Therefore, it is highly likely that the beneficial effects of lipoaspirate post fat grafting procedures are a result of this adipogenic niche and cells. Numerous technical advancements in the field have allowed for modifications in the protocol to further process lipoaspirate into microfat and nanofat thereby making the use of smaller gauge needles possible for fat injections to correct volume losses created by aging or diseases for softer more delicate areas. Microfat is generated using similar approaches as generating fat but using smaller multiport cannula [207]. Nanofat is generated by further processing microfat by mechanical emulsification using syringes leaving fine emulsified fat. Nanofat is generally used for injections using a 27 g needle for correcting delicate areas such as superficial rhytides, scars, eyelids and dark under-eye areas. Recent advances in techniques leading to combining routine fat grafting with nanofat may provide better outcomes for patients with problematic wound management where traditional fat grafts are unable to deliver desired clinical outcomes [208].

While autologous fat grafting is shown in numerous trials to be a safe and easy to perform technique (see Table 2), there are challenges that still remain. These challenges are associated with fat graft survival, necrosis, and loss in volume. Recently, it has been reported that clinical applications using mixed purified/processed fat with 50% or more of autologous platelet rich plasma (PRP) greatly improved fat grafting outcomes and it was no surprise that the improvement was contributed to better survival of fat cells and ADSCs differentiation triggered by the potent growth factors present in PRP such as PDGF, TGFβ, VEGF and EGF [209].

Many clinical applications have also tapped into the therapeutic properties of SVF considering SVF heterogeneous cellular composition. Mechanistically, multiple cell types of SVF might work together to provide a reconstituted niche where numerous signaling pathways listed in Table 1 come together to trigger the efficient healing process and improved therapeutical outcomes [210,211]. SVF and its application as a therapeutic have been extensively reviewed [212,213]. Homogeneous ADSCs isolated from SVF also reported to contribute to efficient regeneration, immunomodulation and wound healing either alone or when mixed in clinical prep with SVF to obtain ADSCs-enriched SVF [179,214]. Many preclinical and clinical studies have demonstrated that the administration of ADSCs improves wound healing by means of the increased accelerated wound closure, reduces scarring, promotes collagen synthesis, angiogenesis, and improves tensile strength. In these lines of clinical trials ADSCs have shown a great potential for the treatment of chronic wounds due to their enhanced growth factors secretion triggering angiogenesis. These factors include VEGF and many others listed in Table 1 and also reviewed by Toyserkani [215].

In addition, ADSCs have been reported to have therapeutic benefit in skin. ADSCs cultured in PRP have been used and are shown to be safe for many clinical applications listed in Table 2, [216]. Ex vivo data demonstrate that ADSCs maintained in PRP-containing media have been shown to have stimulatory effects on the proliferation and migration of dermal fibroblasts and keratinocytes, suggesting that mechanistic explanation of the success in this group of clinical trials might be embedded in ADSCs ability to support re-epithelialization potential by providing guidance and paracrine instructions for maintenance of epidermal organization and homeostasis [133,217,218]. The role of ADSCs in a double paracrine loop provide evidence of an intricate relationships between ADSCs and various cellular components of the dermis and epidermis (Figure 1) and, importantly, signaling pathways involved in skin homeostasis. Taking into consideration ADSCs’ secretory properties, one might suggest that such regulations are probably deployed through the same key growth factors and matrikines that we have listed in Table 1 [11,27]. However, further experiments are required to tease apart the mechanistic underpinning of these highly complex interactions and, importantly, to ensure reliable and reproducible results of these treatments.

## 8. Conclusions

There remain many concerns regarding the use of ADSCs in dermatology, including a lack of mechanistic details on how these cells act as precursors or influence keratinocytes, fibroblasts, and endothelial cells, or as a secretion vehicle of soluble factors. Additionally, while many preclinical and clinical studies have shown that a therapeutic effect results from the use of ADSC transplant or conditioned media, there is no systematic information currently available regarding whether donor age modifies ADSC regenerative or wound healing potential. Even though it has long been known that advanced age is negatively correlated with an organism’s reparative and regenerative potential; scarce, conflicting data that is available concerning the effects of donor age on ADSC function is not sufficient to warrant absence of long term adverse reaction to these cell use. Several ex vivo reports involving various species (rodents, monkeys, and humans) have indicated that aging is accompanied by many changes in biological processes affecting ADSCs (see in greater detail in [23,190,248]). One striking example highlights the importance of such investigations. In the recent study, Kato et al. evaluated whether donor age influences the potential of ADSCs deployed to assist wound healing. The reported data demonstrate that while the treatment with adult’s BM-MSCs or their ADSCs in cutaneous wound promotes better tissue repair/regeneration, efficacy of both of these therapeutic approaches was negatively affected by donor age [33]. It has been suggested that this might be due to changes in the SC paracrine factors secretion. Further studies are necessary to establish the optimal, long-lasting and, importantly, safe strategy for ADSCs applications in the treatment of skin pathological conditions and aging symptoms in patients.

## Figures and Tables

**Table 1:** Growth factors and their function in skin homeostasis, regeneration and repair.

Growth Factor	Primary Target Cells and Effect	References
HB-EGF	Keratinocyte and fibroblast mitogen	[75]
FGFs 1, 2, and 4	Angiogenic and fibroblast mitogen	[76,77]
PDGF	Chemotactic for macrophages, fibroblasts; macrophage activation; fibroblast mitogen, and matrix production	[78]
IGF-1	Endothelial cell fibroblast mitogen	[79,80]
TGF-β1 and β2	Keratinocyte migration; chemotactic for macrophages and fibroblasts	[81]
TGF-β3	Wounded mouse skin, cutaneous rat wounds; Antiscarring	[82,83]
IL-1α and –β	Early activators of growth factor expression in macrophages, keratinocytes and fibroblasts; function in wound healing	[84,85]
IL-10	Regenerative wound repair, Optimization of fibroblast and endothelial progenitor cells (EPC) function in differentiation, Attenuation of inflammatory response	[86,87,88,89]
IL-4	Induction of fibroblast migration and differentiation, ECM production, control of MMP, Lower dermis maintenance, Wound healing	[90,91,92,93,94]
IL-12	Epithelial cells under mouse tongue; Early inflammatory response and vascularization in wound healing, Regulation of Growth factors synthesis	[95]
IL-6, IL-8, IL-17a	Promote connection between immune cells and keratinocytes, fibroblasts Pro-inflammatory response, All the stages of wound healing,	[96,97]
LEP and LEPR	Keratinocytes proliferation, fibroblasts migration, Hyaluronic acid and collagen synthesis, Re-epithelialization processes	[32,98]
Endoglin	Human skin, lung and liver; Co-receptor for TGF-β, Promotes wound healing	[99]
Adipoq	Recruitment of fibroblasts and endothelial cells; Dermis regeneration, Wound healing, Suppression of inflammation	[32,100]
IGFBP1, IGFBP3	Human dermal fibroblasts, human skin Collagen synthesis, Elastogenesis in dermal fibroblasts, Angiogenesis, Proliferation and differentiation	[101,102,103]
CSF1, CSF3 and receptor CSFR1	Rat dermis Stimulation of TGFβ, Anti-inflammatory, Wound healing	Patent US8273712 B2 [104]
PPBP/NAP-2	Fibroblast; mitogenic activity, Modulation of immune cells	[103,105]
HGF	Keratinocytes migration and differentiation; Scarless wound healing	[30,106,107]
NGFR	Human hair follicle cells, ECM remodeling, Wound healing, Modulation of immune cells	[108]
EGF	Rabbit skin cells, White pig skin, human fibroblasts, rat skin, Activation of dormant stem cells, Wound healing, Skin rejuvenation, Production of collagen, elastin, ECM remodeling	[33,109,110,111]
TNF-α	Mice epithelial cells, Similar to the IL-1	[84]
Activin A and Activin B	Rat; Cutaneous wound healing, Human adipose progenitors proliferation and differentiation	[112,113,114,115]

**Table 2:** ADSCs in Clinical Trials for Dermatological Applications.

Condition	Title	Trial Number	Status	References
Scars	A Phase I/II Study of Autologous Fat Transfer for Scar Prevention and Remodeling.	NCT01119326	REGISTERED	[219,220]
	A Phase II/III Clinical Study of Autologous Cultured Adipocytes (ANTG-adip) for the Treatment of Depressed Scar to Evaluate Safety and Efficacy.	NCT00992147	COMPLETED	
	A Phase I Open-labeled, Single-arm, Single-centred Study to Test the Safety of ADSC-SVF-002 in Subjects With Soft Tissue Defects or Abnormal Wound Healing.	NCT02590042	RECRUITING	
Facial Atrophy	A Phase II Double-blind, Randomized, Study to Assess the Efficacy of Facial Fat Grafts Supplemented With Autologous, Adipose Derived Stromal Vascular Fraction (SVF).	NCT02526576	RECRUITING	[210,221,222,223]
	A Phase One, Open Label, Single Arm Study to Demonstrate the Safety of Antria Cell Preparation Process During Facial Fat Grafting Assisted With Autologous, Adipose Derived Stromal Vascular Fraction (SVF).	NCT01828723	COMPLETED	
	Preliminary Investigation of the Effect of Human Adipose Tissue-derived Mesenchymal Stem Cell (MSC) in Progressive Hemifacial Atrophy (Romberg’s Disease).	NCT01309061	COMPLETED	
	Potential of Mesenchymal Stem Cell Enriched Adipose Tissue Grafting for Contour Deformities of Face.	NCT02494752	REGISTERED	
Burn and wound healing	A Phase 1 Clinical Study to Evaluate the Safety of Allogeneic Adipose-derived Stem Cells in the Subjects With Deep Second-degree Burn Wound.	NCT02394873	COMPLETED	[208,224,225,226]
	Evaluation of Tissue Regeneration Potential (in the Skin) of Child’s Adipose Cells During the Development.	NCT02779205	COMPLETED	
	Feasibility of Obtaining Adipose Derived Regenerative Cells (ADRCs) From Discarded Thermal Burn Eschar Tissue Using Investigational Celution^®^ System for Autologous Treatment of Thermal Burn Injury (The FAST Trial).	NCT02362386	COMPLETED	
Skin graft	Phase 1/2 Study of Autologous Stromal Vascular Fraction in Adipose Tissue Transplantation in Improving Skin Grafting.	NCT02546882	RECRUITING	[227,228,229]
	A Phase I, Two Armed, Open, Prospective and Multicentre Study to Evaluate the Safety of Autologous Tissue-engineered Dermal Substitutes and Dermo-epidermal Skin Substitutes for the Treatment of Large Deep Partial and Full Thickness Skin Defects in Children and Adults.	NCT02145130	RECRUITING	
Reconstructive surgery	Immunophenotyping of Fresh Stromal Vascular Fraction From Adipose Derived Stem Cells (ADSC) Enriched Fat Grafts for Refinements of Reconstructed Breasts.	NCT01771913	COMPLETED	[230,231,232,233,234,235,236,237,238,239]
	Skin improvement in Breast.	NCT01801878	REGISTERED	
	Adipose Stromal Cell Enriched Autologous Fat Grafting for Treating Pain at Amputation Sites: A Single Center Site, Prospective, Randomized, Pilot Outcomes Trial.	NCT02076022	RECRUITING	
	Enriched Autologous Fat Grafting for Treating Pain at Amputation Sites.	NCT01645722	REGISTERED	
	Structural Fat Grafting for Craniofacial Trauma: Effect of Concentrating Endogenous Stromal Cells in the Fat Graft.	NCT01564524	REGISTERED	
	Outcomes After Centrifugation Versus PureGraft for Fatgrafting to the Breast After Breast-conserving Therapy.	NCT01979757	COMPLETED	
	A Clinical Evaluation Of Adipose Derived Regenerative Cells In The Treatment Of Patients With Breast Deformities Post Segmental Breast Resection (Lumpectomy) With Or Without Radiation Therapy. A Phase IV Post Market Study.	NCT00616135	COMPLETED	
Alopecia	Subcutaneous Transplantation of Autologous Cell Enriched Adipose Tissue For Follicular Niche Stimulation in Early Stage Alopecia Androgenetica (STYLE): a Randomized, Blinded, Controlled Trial.	NCT02503852	RECRUITING	[137,240]
	Adipose-derived Stromal Vascular Fraction (SVF) Injections to Stimulate Hair Regrowth for Androgenetic Alopecia.	NCT02626780	REGISTERED	
	Biocellular Regenerative Therapy in Hair Loss: Use of High Density Platelet-Rich Plasma Concentrates and Cell-Enriched Emulsified Adipose-Derived Tissue Stromal Vascular Fraction.	NCT02849470	RECRUITING	
	The Effect of Allogeneic Human Adipose Derived Stem Cell Component Extract on Androgenic Alopecia.	NCT02594046	REGISTERED	
	Point-of-Care Adipose-derived Cells for Hair Growth.	NCT02729415	REGISTERED	
	Adipose Tissue Derived Stem Cell Based Hair Restoration Therapy for Alopecia.	NCT02865421	REGISTERED	
Urticaria or Scleroderma	Experimental Autologous Mesenchymal Stem Cell Therapy in Treatment of Chronic Autoimmune Urticaria.	NCT02824393	REGISTERED	[241,242,243]
	Scleroderma Treatment With Celution Processed Adipose Derived Regenerative Cells (ADRCs) Registry.	NCT02328625	WITHDRAWN	
	Scleroderma Treatment With Celution Processed Adipose Derived Regenerative Cells [134]: A Randomized, Double-Blind, Placebo-Controlled Trial With Incomplete Crossover.	NCT02396238	REGISTERED	
	Subcutaneous Injection of Autologous Adipose Tissue-derived SVF Into the Fingers of Patients With Systemic Sclerosis: Controlled Clinical Trial With Efficacy Assessment.	NCT02558543	RECRUITING	
Pressure Ulcer	Treatment of Hypertensive Leg Ulcer by Adipose Tissue Grafting.	NCT01932021	REGISTERED	[133,244,245,246]
	A Pilot Study: Evaluating the Safety/Feasibility of ADSC on Adults With Stage III or IV Pressure Ulcers.	NCT02375802	RECRUITING	
	Adipose Derived Regenerative Cellular Therapy of Chronic Wounds.	NCT02092870	RECRUITING	
Lipodystrophies	Phase I Study of a Filler Agent Composed of Mesenchymal Stem Cells Obtained From Autologous Adipose Tissue Associated With Hyaluronic Acid.	NCT02034786	REGISTERED	[247]

Source: https://clinicaltrials.gov.
